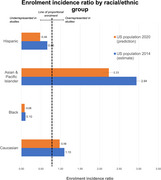# Demographic representation in Phase III clinical trials of anti‐amyloid therapy for Alzheimer’s disease: a cross‐sectional analysis

**DOI:** 10.1002/alz70859_096135

**Published:** 2025-12-25

**Authors:** Justine Tin Nok Chan, Saeed Kayhanian

**Affiliations:** ^1^ School of Clinical Medicine, University of Cambridge, CB2 0SP, Cambridgeshire United Kingdom; ^2^ University of Cambridge, Cambridge, Cambridgeshire United Kingdom

## Abstract

**Background:**

Alzheimer’s disease (AD) is known to vary in prevalence amongst different ethnic and racial groups. Phase III clinical trials set the standard for safety and efficacy of new medicinal therapies. It is therefore essential that recruitment to such trials accurately represents the demographic characteristics of the population at risk. We investigate whether the Phase III studies of anti‐amyloid therapy for AD to date have recruited adequately from different racial groups to reflect this difference in demographic prevalence.

**Method:**

A cross‐sectional analysis of all completed Phase III studies of anti‐amyloid monoclonal antibody therapy for AD. Trial demographic data was extracted and compared to reference data for US disease prevalence by demographic, to generate an Enrollment Incidence Ratio (EIR).

**Result:**

19 trials were identified, with complete demographic data available for 18 of these 19 trials. 19613 patients were included in this analysis. Trials examined seven different drugs or drug combinations of mAbs. There has been no statistically significant change in the proportions of Asian, Hispanic or Black participants in these trials when examined across time. The majority of trial participants were Caucasian persons (83.20%), followed by Asian individuals (9.62%). Native Hawaiian/Pacific Islanders formed the lowest proportion of trial participants (0.02%). The White population was found to be overrepresented in trial populations (EIR = 1.10, 95% CI 1.09‐1.11) whereas Black (EIR = 0.10, 95% CI 0.09‐0.12) and Hispanic individuals (EIR = 0.66, 95% CI 0.62‐0.70) are both significantly underrepresented as compared to US prevalence data (Figure 1).

**Conclusion:**

Our results suggest that Caucasian individuals are overrepresented, while Black and Hispanic individuals are underrepresented in all anti‐amyloid trials for Alzheimer’s disease to date. The current evidence base for anti‐amyloid therapy for AD does not proportionately reflect the racial and ethnic demographics of disease burden. Future trials should aim to proactively recruit from underrepresented racial groups to ensure these drugs are demonstrated as safe and effective in the whole spectrum of the patient population.